# *Dendrobium officinale* nanovesicles: transcriptomic landscape and anti-inflammatory roles

**DOI:** 10.3389/fbioe.2026.1848009

**Published:** 2026-07-29

**Authors:** Biren Wang, Yang Yang, Shuya Zhang, Jiazhen Wu, Yuyan Wei, Songzhu Xu, Yun Li, Qinhua Chen, Guangyi Yang

**Affiliations:** 1 Shenzhen Clinical College of Integrated Chinese and Western Medicine, Guangzhou University of Chinese Medicine, Shenzhen, Guangdong, China; 2 Shenzhen Bao’an Authentic TCM Therapy Hospital, Guangzhou University of Chinese Medicine, Shenzhen, Guangdong, China; 3 School of Pharmacy, Xi’an Jiaotong University Health Science Center, Shanxi, China; 4 Shenzhen Traditional Chinese Medicine Hospital Department of Pharmacy, The Fourth Clinical Medical College of Guangzhou University of Chinese Medicine, Shenzhen, China

**Keywords:** anti-inflammatory roles, coding and non-coding RNAs, comprehensive transcriptomic, dendrobium officinale, extracellular vesicle-like nanoparticles

## Abstract

**Introduction:**

Plant-derived extracellular vesicle-like nanoparticles (EVLNs) represent a promising category of natural nanocarriers with significant potential for therapeutic applications. Nevertheless, the physicochemical properties and pharmacological functions of vesicles derived from Dendrobium officinale (Do-EVLNs) remain inadequately characterized. This study systematically elucidates the structural characteristics, *in vitro* regenerative and anti-inflammatory functions, and the comprehensive whole-transcriptome molecular landscape of Do-EVLNs.

**Methods:**

Do-EVLNs were isolated through differential ultracentrifugation. Physicochemical characterization was conducted to detect vesicle properties. *In vitro* cellular assays were performed to evaluate cellular uptake, biological function of Do-EVLNs in human skin cells. Lipopolysaccharide (LPS)-stimulated RAW 264.7 macrophages were used for *in vitro* anti-inflammatory assessment. Comprehensive strand-specific transcriptomic profiling (miRNAs, lncRNAs, mRNAs, and circRNAs) and differential expression analysis together with bioinformatic functional enrichment analyses were carried out.

**Results:**

Physicochemical characterization revealed spherical vesicles with a mean diameter of 132.1 ± 1.9 nm, a stable yield of 5.7 × 10^10^ particles/mL, and a negative zeta potential of −24 ± 0.16 mV. *In vitro* cellular assays confirmed efficient internalization of Do-EVLNs by key human-skin-resident cells (HUVECs, HACAT cells, and BJ-1 cells). Functionally, Do-EVLNs demonstrated excellent biocompatibility and significantly enhanced tissue repair phenotypes, notably improving the wound closure rate by over 40% within 24 h (p < 0.05), stimulating capillary-like network formation, and reducing apoptosis through the promotion of the G1-to-S cell-cycle transition. Importantly, *in vitro* anti-inflammatory assessment in lipopolysaccharide (LPS)-stimulated RAW 264.7 macrophages indicated that Do-EVLNs exhibited no cytotoxicity at concentrations of 5–20 μg/mL. Upon treatment, Do-EVLNs substantially suppressed inflammation by reversing the transcriptional and translational expression of interleukin-6 (IL-6) in a dose-dependent manner, returning protein secretion to levels approaching those of the baseline vehicle control, while concurrently promoting the upregulation of the anti-inflammatory cytokine IL-10. Comprehensive strand-specific transcriptomic profiling uncovered a highly selective nucleic acid packaging mechanism unique to the EVLNs, distinct from parental tissues. Differential expression analysis identified an enriched and complex cargo in Do-EVLNs, including 1,163 upregulated miRNAs (notably inflammation-resolving markers such as miR-223–5p and the miR-169 family), 2,188 lncRNAs, and 5,505 mRNAs (p < 0.05). Bioinformatic functional enrichment analyses suggested that these specialized molecular cargoes are likely to modulate critical host signaling pathways, particularly the PI3K/AKT pathway, cellular metabolism, and cytoskeletal remodeling, which are crucial in mediating inflammatory responses.

**Discussion:**

These quantitative findings lay a solid foundation for the upcoming development of Do-EVLNs as novel bioactive nanotherapeutics aimed at promoting skin homeostasis and enhancing wound healing.

## 1 Introduction

Plant-derived extracellular vesicle-like nanoparticles (EVLNs) are nanoscale, membrane-bound vesicles secreted by plant cells. They play a critical role in mediating intercellular communication and physiological processes by transporting a variety of bioactive substances, including proteins, lipids, and various RNA species (Teng et al., 2025; Zhao et al., 2024; Kim et al., 2022; Liu et al., 2024). Like mammalian extracellular vesicles (EVs), EVLNs have garnered significant attention in nanomedicine due to their inherent biocompatibility, low immunogenicity, and strong ability to circumvent biological barriers (Nemati et al., 2022). Recent studies have highlighted the preliminary therapeutic potential of vesicles isolated from edible and medicinal plants such as *Zingiber officinale* (Yan et al., 2025), *Vitis vinifera* (Teng et al., 2022), and *Citrus limon* (Bruno et al., 2021) in modulating host immune responses and restoring cellular homeostasis.

Compared to conventional mammalian cell-derived vesicles, plant-derived platforms offer unique and significant advantages for biotherapeutic applications. On one hand, plant systems act as cost-effective and scalable “green bio-factories,” eliminating the need for expensive media and stringent culture conditions required by mammalian cells (De Marchis et al., 2024). On the other hand, because plant cells do not support the replication of human pathogens, materials derived from plants present a substantially lower risk of transmitting zoonotic viruses, *Mycoplasma*, or prions, thus ensuring a superior biosafety profile for clinical and dermatological applications (Bellucci et al., 2021).

Despite their promising therapeutic potential, the field of plant-derived EVLNs remains in its infancy, marked by ongoing controversies and methodological challenges. A primary point of debate lies in the biogenesis and nomenclature of these vesicles. The secretory pathways in plants are not as thoroughly elucidated as the endosomal origins of mammalian exosomes, leading to a growing preference for more rigorous terms such as “exosome-like nanoparticles” or “extracellular vesicle-like nanoparticles.” Additionally, significant methodological hurdles complicate the isolation of EVLNs. Unlike mammalian cell cultures, isolating vesicles from robust plant tissue typically requires mechanical disruption (e.g., juicing or grinding). This process inherently risks the co-isolation of intracellular organelle fragments, making it particularly challenging to differentiate actively secreted vesicles from artificial nanovesicles or cellular debris. Recent methodological investigations have indicated that these rigorous mechanical extraction processes inevitably introduce complex matrix contaminants, rendering downstream purification significantly more difficult than that of mammalian vesicles (Maricchiolo et al., 2026). As a result, achieving standardized extraction protocols and high-purity preparations remains a major challenge, necessitating rigorous physicochemical and omics characterizations.

*Dendrobium officinale* Kimura & Migo, a highly regarded orchid species in traditional Chinese medicine (TCM), is esteemed for its wide range of pharmacological activities, particularly its potent immunomodulatory and anti-inflammatory effects (Tang et al., 2017; Xu et al., 2022). While the primary phytochemical constituents, such as polysaccharides and alkaloids, have been extensively characterized, the existence and functional potential of its endogenously derived EVLNs remain largely unexplored. The complex molecular cargo, particularly the diverse RNA landscape (miRNAs, lncRNAs, mRNAs, and circRNAs) encapsulated within these *D. officinale*-derived EVLNs (Do-EVLNs), may act as primary effectors of the plant’s biological efficacy. Therefore, elucidating the interactions between these nanovesicles and human skin-resident cells—specifically their internalization and subsequent effects on cell viability, migration, and apoptosis—is essential for assessing their translational potential in regenerative medicine and dermatology.

This study addresses existing knowledge gaps by employing a comprehensive transcriptomic approach to systematically profile the diverse RNA landscape of Do-EVLNs. In addition to standardized isolation and physicochemical characterization, the study investigated cellular uptake kinetics and *in vitro* regenerative bioactivity across multiple human skin-resident cell lines (HUVEC, HACAT, and BJ-1). To validate their immunomodulatory potential, the *in vitro* anti-inflammatory efficacy of Do-EVLNs was evaluated using lipopolysaccharide (LPS)-activated RAW 264.7 macrophages. The results demonstrated that Do-EVLNs exhibited excellent biosafety with no detectable cytotoxicity at 5–20 μg/mL and effectively exerted potent anti-inflammatory activities by suppressing the transcription and translation of interleukin-6 (IL-6) in a dose-dependent manner while also facilitating the upregulation of interleukin-10 (IL-10). Furthermore, through high-throughput sequencing of the complete RNA spectrum, the study identified key differentially expressed miRNAs and predicted their functional targets involved in host regulation. The findings presented herein aim to provide a concrete conceptual framework for future research on Do-EVLNs, while offering valuable methodological insights for studies involving plant-derived EVLNs in general.

## 2 Materials and methods

### 2.1 Pretreatment of *Dendrobium officinale*

Fresh specimens of *D. officinale* (n = 18, from three independent biological batches) were collected from their natural habitats in Guangdong Province, China, with uniform developmental stages selected for analysis. Tissue samples were immediately flash-frozen in liquid nitrogen and stored at −80 °C. Botanical authentication was conducted by Prof. Qinhua Chen from the Department of Pharmacy at Shenzhen Traditional Chinese Medicine Hospital, Fourth Clinical Medical College of Guangzhou University of Traditional Chinese Medicine. A voucher specimen (No. 20231,202) was deposited at the herbarium of the Traditional Chinese Medicine Research and Application Center, Shenzhen Pure TCM Treatment Hospital. Subsequently, fresh roots and stems (approximately 398 g) were rinsed with deionized water and homogenized in pre-cooled phosphate-buffered saline (PBS; Sangon Biotech, Cat. No. E607008) at a defined weight-to-volume ratio. The homogenate underwent differential centrifugation at 4 °C to remove cellular debris and larger impurities.

### 2.2 Isolation of Do-EVLNs

Do-EVLNs were isolated from the fresh *D. officinale* using differential ultracentrifugation, following previously established methods with minor modifications (Cai et al., 2025). Plant tissue was homogenized in PBS using a high-speed blender for 10 min at 4 °C. The homogenate was then filtered through six layers of medical gauze. To eliminate cellular debris and large aggregates, the filtrate was sequentially centrifuged at 500 × g for 10 min, 2,000 × g for 20 min, and 10,000 × g for 60 min at 4 °C. The resulting supernatant was ultracentrifuged at 100,000 × g for 70 min at 4 °C using a Thermo Sorvall WX 90+ ultracentrifuge equipped with an appropriate rotor (Thermo Fisher Scientific, Waltham, MA, United States). The pellet was resuspended in sterile PBS, filtered through a 0.22 μm syringe filter (Millipore, Billerica, MA, United States), and designated as Do-EVLNs. The purified vesicles were either used immediately or aliquoted and stored at −80 °C.

### 2.3 Transmission electron microscopy

The morphology of Do-EVLNs was characterized using transmission electron microscopy (TEM). A 20 µL aliquot of the suspension was pipetted onto a Formvar/carbon-coated copper grid and incubated at room temperature for 1 min. Excess liquid was blotted using ashless filter paper. Subsequently, the grid was negatively stained with 20 µL of 2% (w/v) uranyl acetate (Xinrui Biotechnology, Zhengzhou, China) for 1 min. After removing the residual stain and allowing the samples to air-dry, imaging was performed using an HT-7700 transmission electron microscope (Hitachi, Tokyo, Japan) at an accelerating voltage of 80 kV.

### 2.4 Nanoparticle tracking analysis

To determine size distribution and particle concentration, frozen Do-EVLN aliquots were thawed at 25 °C, maintained on ice, and appropriately diluted in 0.22 µm-filtered PBS. Nanoparticle tracking analysis (NTA) was conducted using a ZetaView PMX 110 instrument (Particle Metrix, Meerbusch, Germany). The hydrodynamic diameter and particle concentration were subsequently analyzed using the integrated ZetaView software.

### 2.5 *In vitro* cellular uptake and cytotoxicity assessment of Do-EVLNs

#### 2.5.1 Definition and preparation of control groups

To assess the biological efficacy of purified Do-EVLNs, two independent control groups were established for all cellular assays: a vehicle control (denoted as “control”), consisting of sterile PBS applied at a volume equivalent to the treatment groups, and a whole plant extract control (designated as “5% extract”). The 5% extract control was prepared by homogenizing fresh *D. officinale* tissue (Do-tissue), filtering the homogenate through a 0.22 µm membrane, and supplementing the crude filtrate into the culture medium at a 1:19 volume ratio (v/v), resulting in a final concentration of 5% (50 µL of filtrate per 950 µL of medium). This whole-extract control served as a comparative benchmark to distinguish the specific therapeutic effects of encapsulated nanoscale vesicles from those exerted by the raw, soluble phytochemical constituents of the plant.

#### 2.5.2 Cellular internalization assay

To evaluate the cellular uptake of Do-EVLNs, the vesicles were labeled with a lipophilic fluorescent dye and purified via ultracentrifugation to eliminate unbound dye. HACAT, HUVEC, and BJ-1 cells were seeded onto glass coverslips and incubated with labeled Do-EVLNs (20 μg/mL) for 24 h. Cells treated with an equivalent volume of PBS served as a negative background reference. Following incubation, cells were fixed with 4% paraformaldehyde, and nuclei were counterstained with DAPI. Intracellular localization and uptake efficiency were analyzed using laser scanning confocal microscopy (LSCM).

#### 2.5.3 Cytotoxicity assessment

Cell viability was evaluated via the Cell Counting Kit-8 (CCK-8) assay (Dojindo, Kumamoto, Japan). HACAT cells and HUVECs were seeded into 96-well plates, cultured overnight, and subsequently treated with varying concentrations of Do-EVLNs (5, 10, 20, 40, and 100 μg/mL), the 5% extract control, or the PBS vehicle for 48 h. Following the manufacturer’s instructions, absorbance was measured at 450 nm to assess cytotoxic effects, comparative proliferative capacities, and to establish optimal working concentrations.

#### 2.5.4 Tube formation assay

The angiogenic activity of Do-EVLNs was assessed using a Matrigel-based tube formation assay. Pre-chilled 96-well plates were coated with Matrigel (Corning, New York, NY, United States) and incubated at 37 °C for 30 min to facilitate polymerization. HUVECs were then seeded onto the Matrigel and treated with specified concentrations of Do-EVLNs, the 5% extract control, or the PBS vehicle, maintaining uniform treatment volumes across all groups. Capillary-like network formation was observed and photographed at 0, 6, and 12 h post-seeding. Total tube length and the number of junctions were quantified using ImageJ software (version 1.54).

#### 2.5.5 Wound-healing assay

Cell migration was evaluated using a scratch wound healing assay. HACAT cells and HUVECs (2 × 10^5^ cells/well) were cultured in 6-well plates until reaching approximately 90% confluence. A linear scratch was generated using a sterile 20 μL pipette tip. Following three washes with PBS to remove cellular debris, the cells were incubated in low-serum medium containing 1% fetal bovine serum (FBS) and supplemented with either the indicated concentrations of Do-EVLNs, the 5% Extract control, or the PBS vehicle under equalized volume conditions. Wound closure was photographed at 0, 24, and 48 h post-scratching using an inverted microscope. The wound closure rate, defined as the percentage of wound area reduction, was quantified using ImageJ software.

#### 2.5.6 Cell cycle and apoptosis analysis

HACAT cells and HUVECs were treated with Do-EVLNs, the 5% extract control, or the PBS vehicle under equal volume conditions for 48 h. For cell cycle profiling, cells were harvested, washed with cold PBS, and fixed in 70% ice-cold ethanol at −20 °C overnight. Fixed cells were then stained with a propidium iodide (PI)/RNase A solution (Beyotime, China) for 30 min at 37 °C in the dark, with cell cycle distribution analyzed via flow cytometry. Apoptosis was quantified using an Annexin V-APC/PI dual staining kit. Treated cells were resuspended in binding buffer and stained with Annexin V-APC and PI for 15 min at room temperature in the dark. Samples were analyzed within 1 h to differentiate early apoptotic (Annexin V^+^/PI^−^) and late apoptotic/necrotic (Annexin V^+^/PI^+^) populations. Flow cytometric data were processed using FlowJo software.

### 2.6 *In vitro* anti-inflammatory assessment

#### 2.6.1 Cell culture, passaging, and cryopreservation of RAW 264.7 cells

Mouse macrophage leukemia cells (RAW 264.7) were maintained in Dulbecco’s modified Eagle medium (DMEM; AstroBio, China) supplemented with 10% FBS (AstroBio, China) at 37 °C under a humidified atmosphere containing 5% CO_2_. Cryopreserved cells were rapidly thawed in a 37 °C water bath and subsequently centrifuged to remove residual dimethyl sulfoxide (DMSO) prior to seeding. Upon reaching 80%–90% confluence, cells were detached using 0.25% trypsin–EDTA (Vazyme, China) and passaged at splitting ratios ranging from 1:3 to 1:6. For long-term preservation, cells in the logarithmic growth phase were harvested, resuspended in pre-cooled freezing medium, and subjected to a stepwise cooling protocol (4 °C for 30 min, −20 °C for 2 h, and −80 °C overnight) before final transfer to liquid nitrogen.

#### 2.6.2 Lipopolysaccharide-induced inflammatory model in RAW264.7 cells

To establish the *in vitro* inflammation model, logarithmic-phase RAW 264.7 cells were detached, neutralized, and harvested via centrifugation as described. The cell pellet was resuspended in complete culture medium, quantified using a hemocytometer, and adjusted to a final density of 4 × 10^5^ cells/mL. The cell suspension was then seeded into culture plates and incubated until reaching approximately 80% confluence. Following this, the culture medium was replaced with fresh complete medium supplemented with 1 μg/mL LPS, and the cells were incubated for an additional 24 h to induce the macrophage inflammation model.

#### 2.6.3 Cytotoxicity and cell viability assessment on RAW 264.7

To eliminate potential confounding effects of cytotoxicity on the cellular inflammatory response, cell viability was assessed using a CCK-8 assay (Vazyme, China). RAW 264.7 cells were inoculated into 96-well plates (4 × 10^5^ cells/mL, 100 μL/well) with six parallel replicates per group and incubated for 24 h. Cells were then treated as per the experimental groups with DMEM vehicle control, LPS, 5% Extract, or varying concentrations of Do-EVLNs (5, 10, and 20 μg/mL), followed by an additional 24 h of incubation. At the end of the treatment period, 10 μL of CCK-8 reagent was added to each well. After an hour of incubation at 37 °C under light-protected conditions, absorbance was measured at 450 nm using a microplate reader (Thermo Fisher Scientific, United States) to evaluate cell viability.

#### 2.6.4 Measurement of nitric oxide (NO) secretion

The secretion of NO into the extracellular environment was quantified using a Griess Reagent Nitric Oxide Assay Kit (Beyotime, China). RAW 264.7 macrophages were seeded in 96-well plates at 4 × 10^5^ cells/mL (100 μL/well) and incubated for 24 h. Afterward, the cells were treated with either the DMEM vehicle control, LPS, 5% extract, or varying concentrations of Do-EVLNs (5, 10, and 20 μg/mL) for 12 h. Cell culture supernatants (50 μL/well) were collected and transferred to a new 96-well plate. Sodium nitrite (NaNO_2_) standards were prepared in DMEM to generate a calibration curve ranging from 0 to 100 μM. Subsequently, 50 μL of Griess Reagent I and 50 μL of Griess Reagent II (both equilibrated to room temperature) were added to each well. Chromogenic development was measured at a 540 nm wavelength using a microplate reader.

#### 2.6.5 Quantitative real-time PCR analysis of inflammatory genes

Total RNA extraction from treated RAW 264.7 cells (seeded at 4 × 10^5^ cells/mL in 24-well plates and treated for 12 h) was performed using the RISO™ RNA Extraction Reagent (Cwbio, China). Cells were washed twice with ice-cold PBS, lysed in 1 mL of lysis buffer for 5 min at room temperature, and separated with 0.2 mL of chloroform. Following centrifugation at 12,000 × g for 15 min at 4 °C, the aqueous phase was transferred, and RNA was precipitated using 0.5 mL of isopropanol. The resulting RNA pellet was washed with 1 mL of 70% ethanol, centrifuged at 7,000 × g for 5 min at 4 °C, air-dried, and then dissolved in DEPC-treated water at 55 °C–60 °C. RNA integrity was verified by 1.0% agarose gel electrophoresis using a gel imaging system (Bio-Rad, United States).

Real-time PCR amplifications were conducted on an ABI PCR system (Applied Biosystems, United States) using ChamQ Blue Universal SYBR qPCR Master Mix (Vazyme, China). Relative mRNA expression levels of target inflammatory genes (including TNF, IL-6, and IL-10) were normalized against the internal reference gene GAPDH and quantified using the comparative 2^−ΔΔCt^ method.

#### 2.6.6 Enzyme-linked immunosorbent assay (ELISA) for cytokine quantification

The concentrations of key pro-inflammatory and anti-inflammatory cytokines, including TNF-α, TGF-β, IL-6, and IL-10, secreted into the culture supernatant were assessed using specific ELISA kits (LCS Bio, China). Prior to the assay, all reagents and samples were equilibrated to room temperature (25–28 °C) for 60 min. Standard dilutions (50 μL) and experimental supernatants (50 μL) were added to their designated microplate wells, while 50 μL of sample diluent was allocated to the blank control wells.

Following this, 100 μL of horseradish peroxidase (HRP)-conjugated detection antibody was added to each well. The plate was sealed and incubated at 37 °C for 45 min. After discarding the liquid, the wells were washed five times with 350 μL of wash buffer, allowing 20 s of soaking per cycle, and thoroughly blotted dry. Substrates A and B (50 μL each) were added to initiate color development at 37 °C for 15 min in the dark. The reaction was halted by adding 50 μL of stop solution, and optical density (OD values) were determined at 450 nm within 15 min using a microplate reader.

### 2.7 RNA extraction and small RNA sequencing

Total RNA was extracted from Do-tissue and Do-EVLNs using the Plant Total RNA Extraction Kit (Cat. No. DP441; Tiangen Biotech, Beijing, China). RNA integrity and size distribution were assessed via capillary electrophoresis utilizing a Qsep100 Bio-Fragment Analyzer (Bioptic Inc., Taiwan, China) after dilution with NR1 Matching Diluent. Subsequently, miRNAs were specifically isolated using the miRNeasy Mini Kit (Cat. No. 217004; QIAGEN, Hilden, Germany) according to the manufacturer’s protocol and stored at −80 °C for downstream analysis.

Small RNA libraries (n = three biological replicates per group; total of six libraries) were constructed by incorporating unique molecular identifiers (UMIs) during reverse transcription to minimize PCR amplification bias. All library preparation and paired-end high-throughput sequencing (PE150) on the Illumina NextSeq platform were conducted by Wuhan GeneCreate Biological Engineering Co., Ltd (Wuhan, China). The quality of the raw sequencing data was evaluated using FastQC (version 0.11.9).

### 2.8 Sequencing data pre-processing and alignment

Raw sequencing reads were processed with fastp (version 0.23.2) to remove adapters, poly-A tracts, and low-quality reads (Q ≤ 20 or > 10% ambiguous bases). For mRNA, lncRNA, and circRNA analyses, clean reads were mapped to the NCBI *Dendrobium catenatum* reference genome (Genome assembly ASM160598v2) using HISAT2 (version 2.1.0). For small RNA profiling, clean reads were first aligned to the Rfam database (version 14.9) via Bowtie (version 1.3.0) to exclude non-coding RNAs (e.g., rRNAs and tRNAs), filtering out sequences shorter than 20 nt. The retained clean sequences were then aligned against the miRBase database (Release 22) to identify known miRNAs.

### 2.9 Transcript assembly and RNA identification

Transcript abundance was quantified using featureCounts (version 2.0.1) and normalized to fragments per kilobase of transcript per million mapped reads (FPKM). The identification of specific RNA subtypes was conducted as follows.

LncRNAs: transcripts were assembled using StringTie (version 2.1.5) and compared against the reference genome via GffCompare (version 0.11.2). Transcripts longer than 200 nt with an open reading frame (ORF) shorter than 100 amino acids were evaluated for coding potential using CPC2, CNCI, Pfam, and FEELnc. Candidates predicted as non-coding by at least three of these tools were classified as novel lncRNAs.

miRNAs: retained small RNA reads were mapped to mature and precursor sequences in miRBase (release 22.1). Known miRNAs were identified and quantified using miRDeep2, grouped into families, and analyzed for conservation patterns.

CircRNAs: circular RNAs were identified by detecting non-canonical back-splice junctions (BSJs) using an anchor-based alignment strategy via CIRIquant (version 1.1.2). The relative circRNA abundance was calculated based on the circular-to-linear junction ratio using the formula R_junction_ = 2BSJ/(2BSJ + FSJ).

### 2.10 Differential expression analysis

Differential expression analysis across all identified RNA profiles (miRNAs, lncRNAs, mRNAs, and circRNAs) was performed using the DESeq2 R package. Differentially expressed transcripts and non-coding RNAs were stringently defined by a Benjamini–Hochberg (BH)-adjusted P-value < 0.05 and an absolute |log_2_ fold change| ≥ 1.

### 2.11 Target prediction and functional enrichment

To elucidate the biological implications of the Do-EVLN cargo, computational predictions were made for the target genes of the identified differentially expressed transcripts and non-coding RNAs. Subsequently, Gene Ontology (GO) and Kyoto Encyclopedia of Genes and Genomes (KEGG) pathway enrichment analyses were conducted, evaluating their overrepresentation relative to the genomic background using a hypergeometric test. The false discovery rate (FDR) was controlled via the BH method, and terms or pathways with an adjusted P-value < 0.05 were considered significantly enriched.

### 2.12 Quantitative real-time PCR

The relative abundance of target transcripts was quantified via one-step reverse transcription-quantitative PCR (RT-qPCR). Total RNA underwent amplification on a Real-Time PCR System, with all experiments executed using three independent biological replicates, each analyzed through three technical replicates. Specific primers for target genes were designed, employing Species 5.8S RNA as the internal control for normalization. Thermal cycling conditions and reaction chemistry were optimized to maximize amplification efficiency. Post-reaction, quantification cycle (Cq) values were determined using integrated software, ensuring consistent application of thresholds and baselines across all plates. The specificity of amplified products was rigorously confirmed through melting curve analysis to exclude primer dimers and non-specific artifacts. Relative fold changes in gene expression were calculated using the comparative 2^−ΔΔCq^ method. Statistical validation of RT-qPCR results was performed utilizing one-way analysis of variance (ANOVA) in GraphPad Prism software, with a significance threshold of *p* < 0.05.

### 2.13 Statistical analysis

Data analysis was performed using IBM SPSS Statistics software (v22.0), with all biological experiments independently conducted in triplicate. Quantitative results are presented as the mean ± standard error of the mean (SEM). Statistical comparisons between two experimental groups were assessed using the two-tailed Student’s t-test, while multi-group comparisons—including Control, 5% Extract, and varying concentrations of Do-EVLNs—were evaluated via one-way ANOVA followed by Tukey’s honestly significant difference (HSD) *post hoc* test. A stratified probability (*p*-value) system represented by asterisks was applied across all figures and captions to communicate statistical significance clearly: one asterisk (*) indicates *p* < 0.05, two asterisks (**) denote *p* < 0.01, three asterisks (***) represent *p* < 0.001, and four asterisks (****) signify *p* < 0.0001, while differences with *p* ≥ 0.05 were classified as statistically non-significant (labeled as ns). The exact level of statistical significance for each comparison is detailed within the respective figure captions in the “Results” section, and all graphical representations, flow cytometry data charts, and curve fittings were generated using OriginPro (v2017) and GraphPad Prism software.

## 3 Results

### 3.1 Isolation and characterization of Do-EVLNs

**FIGURE 1:**
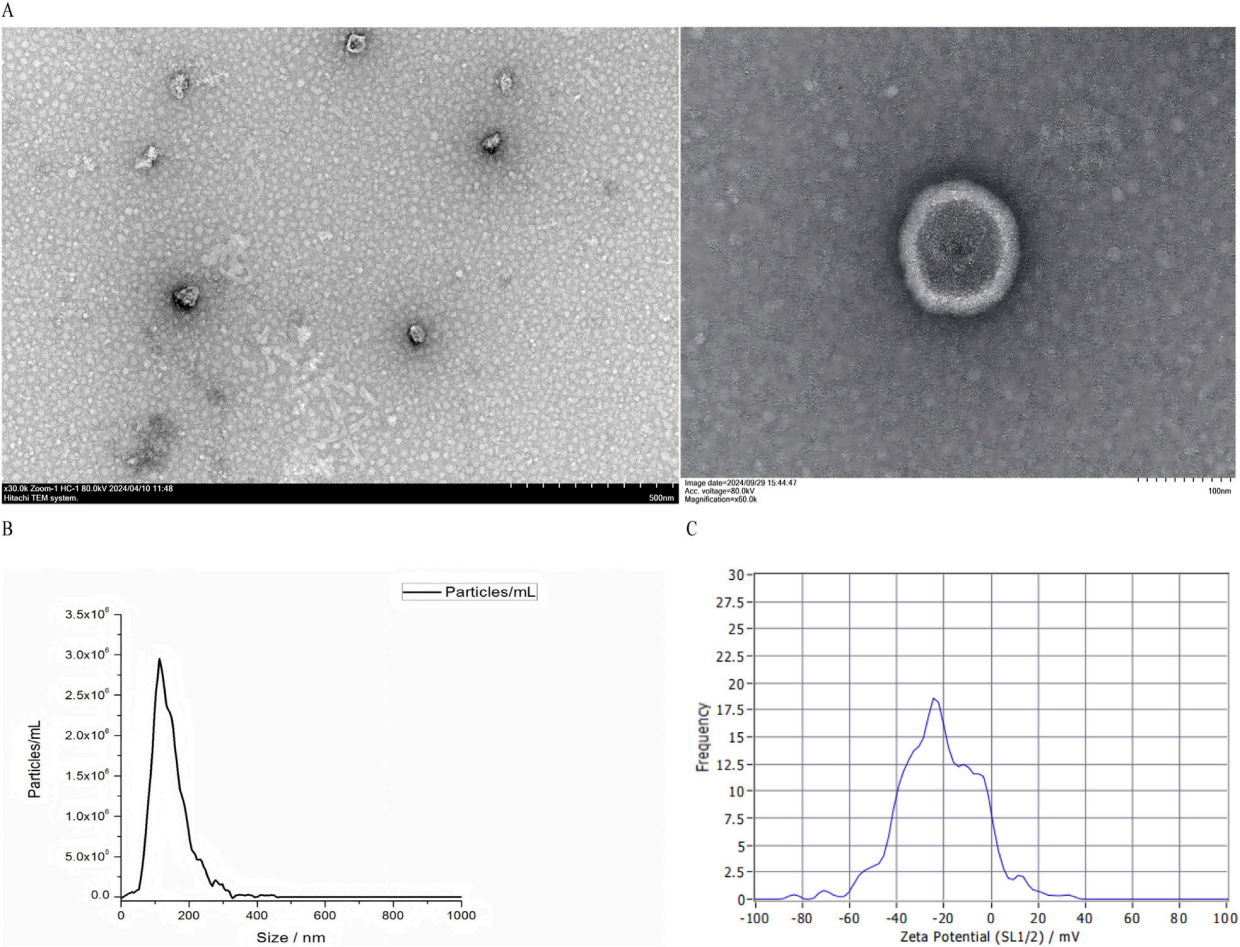
Characterization of Do-EVLNs. **(A)** TEM images of isolated Do-EVLNs. Left low-magnification micrograph shows overall distribution of vesicles (scale bar = 500 nm), and right high-magnification micrograph displays the typical cup-shaped morphology of individual Do-EVLNs (scale bar = 100 nm). **(B)** Size distribution of isolated Do-EVLNs. **(C)** Zeta potential analysis of Do-EVLNs (*p* ≥ 0.05, ns; *p < 0.05, **p < 0.01, ***p < 0.001, and ****p < 0.0001).

The morphological characteristics of the isolated Do-EVLNs were first analyzed using TEM. The micrographs obtained displayed intact, cup-shaped or spherical, membrane-bound vesicles (Figure 1A), confirming the successful isolation of Do-EVLNs from Do-tissue. To further characterize the physicochemical properties of these vesicles, NTA was performed to assess their size distribution and particle concentration. The NTA results indicated a relatively homogeneous population with a mean particle diameter of 132.1 ± 1.9 nm and a concentration of 5.7 × 10^10^ particles/mL (Figure 1B). Furthermore, the surface charge of Do-EVLNs was evaluated, revealing a distinct negative zeta potential of −24 ± 0.16 mV (Figure 1C). These comprehensive physical characterizations highlight the structural integrity, stability, and high yield of Do-EVLNs.

### 3.2 *In vitro* uptake and safety detection of Do-EVLNs

**FIGURE 2:**
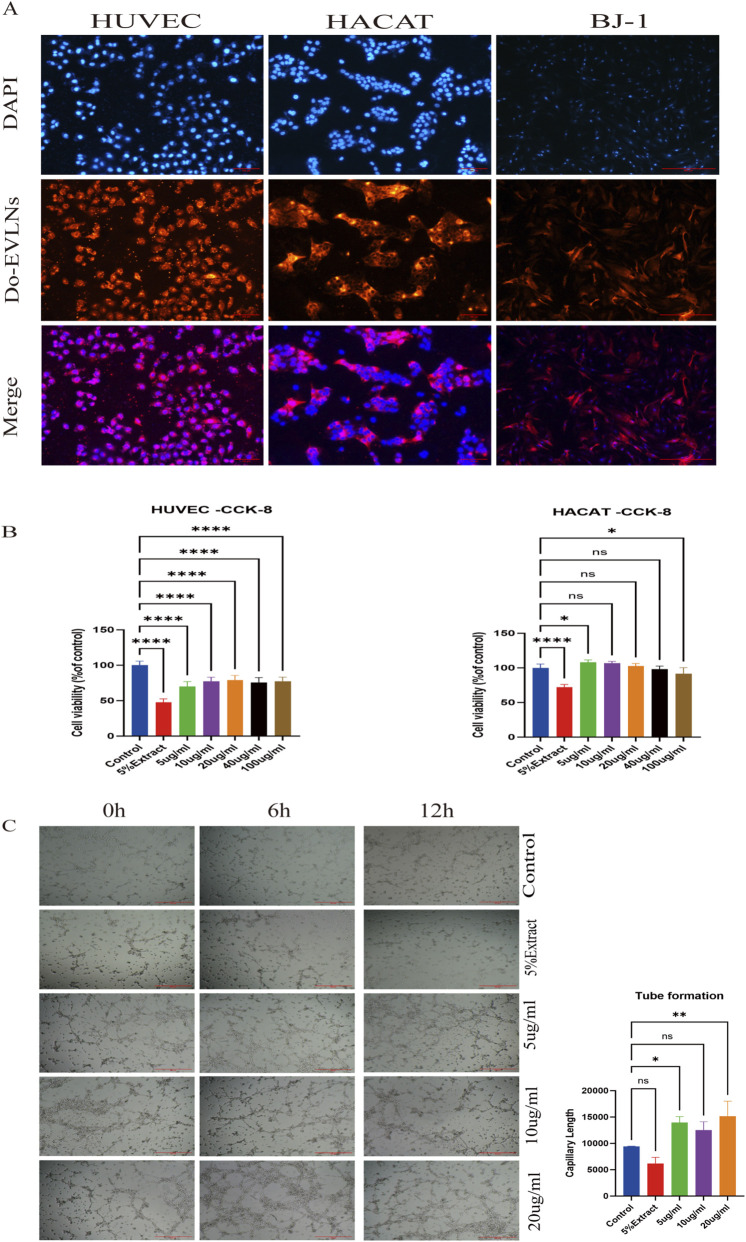
*In vitro* uptake and safety evaluation. **(A)** Uptake of Do-EVLNs by three cell types. Scale bar specifications for each cell group: HUVECs (scale bar = 100 μm); HACAT (scale bar = 100 μm); BJ-1 (scale bar = 500 μm). **(B)** CCK-8 assay showing cell proliferation. **(C)** Tube formation of HUVEC (scale bar = 500 μm) (P ≥ 0.05, ns; *p < 0.05, **p < 0.01, ***p < 0.001, and ****p < 0.0001).

Internalization assays demonstrated that Do-EVLNs were efficiently endocytosed by HUVECs, HACAT cells, and BJ-1 cells (Figure 2A) (Supplementary Figure S3). Regarding cytotoxicity, Do-EVLNs exhibited superior biosafety toward HUVECs compared to the 5% Extract control while also maintaining a favorable safety profile in HACAT cells. This establishes a solid experimental foundation for subsequent functional investigations (Figure 2B). To evaluate angiogenic potential, Matrigel tube formation assays were conducted using HUVECs, revealing that Do-EVLNs significantly stimulated capillary-like network formation, resulting in an increased total tube length compared to the Control group (Figure 2C). These findings suggest a critical role for Do-EVLNs in facilitating vascularization during wound healing. Additionally, scratch-wound healing assays confirmed that Do-EVLN treatment accelerated horizontal migration, achieving an over 40% increase in the wound closure rate at 24 h.

**FIGURE 3:**
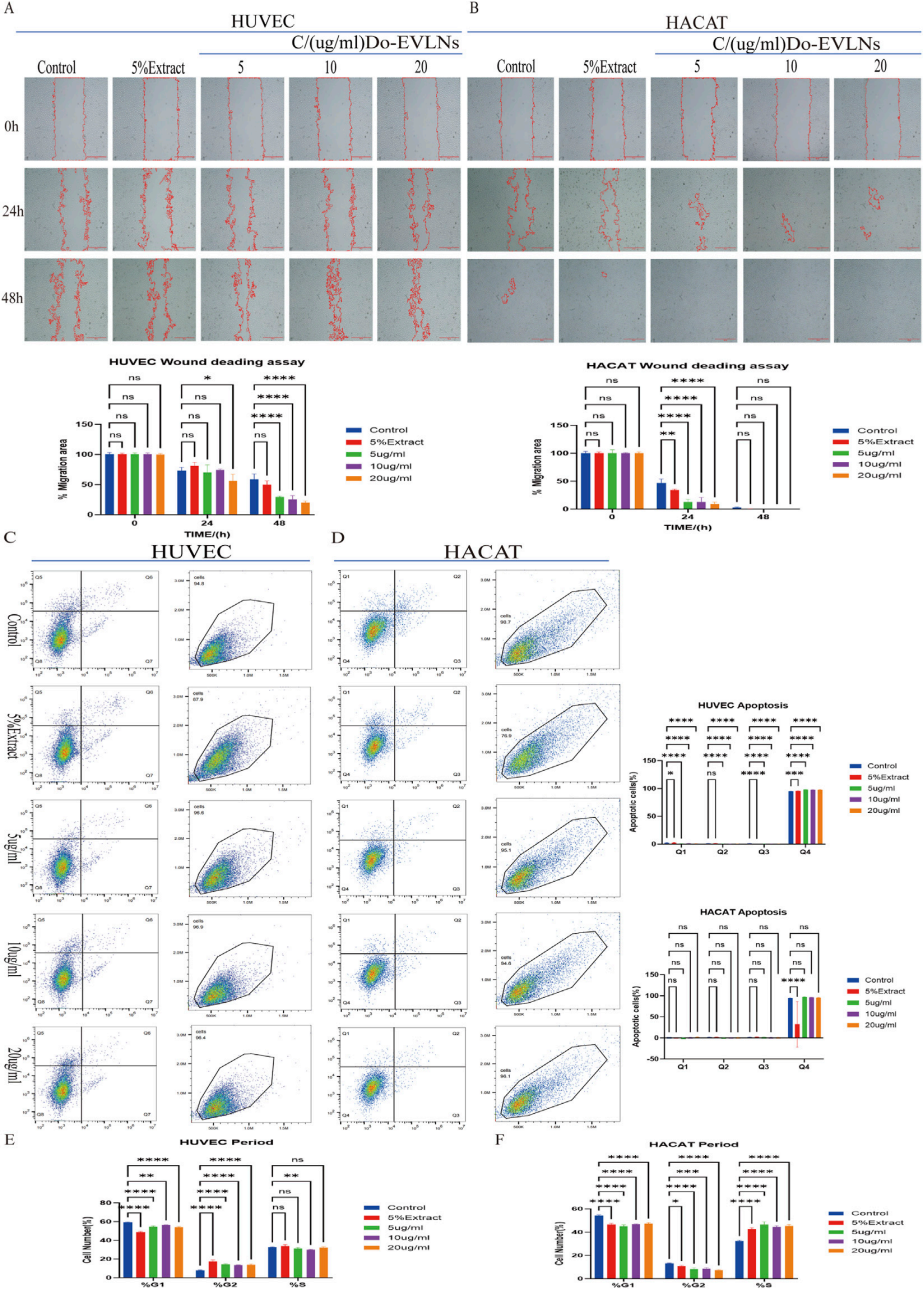
Cell migration, cell cycle, and apoptosis. **(A)** Migration of HUVECs (scale bar = 500 μm). **(B)** Migration of HACAT cells (scale bar = 500 μm). **(C)** Apoptotic changes in HUVECs. **(D)** Apoptotic changes in HACAT cells. **(E)** Cell cycle changes in HUVECs. **(F)** Cell cycle changes in HACAT cells (*p* ≥ 0.05, ns; *p < 0.05, **p < 0.01, ***p < 0.001, and ****p < 0.0001).

Time-course microscopic observations at 0, 24, and 48 h further reinforced that Do-EVLNs across all tested concentrations significantly enhanced the migration of HUVECs and HACAT cells compared to both the Control and the 5% Extract groups (Figures 3A,B). Flow cytometric analysis of Annexin V/PI staining indicated a substantial reduction in apoptotic cell populations in both skin-resident cell types following Do-EVLN administration (Figures 3C,D). Moreover, cell-cycle profiling revealed a shift in the cell population, characterized by a higher proportion of cells in the S phase, with a concomitant decrease in the G2-to-M phase (Figures 3E,F). This S-phase enrichment suggests that Do-EVLNs promote the proliferation of skin-resident cells by accelerating the G1-to-S transition and enhancing mitotic activity.

These findings demonstrate that Do-EVLNs undergo efficient endocytosis and exhibit favorable biosafety profiles in skin-resident cells. These results provide a strong theoretical basis for the application of Do-EVLNs in the field of skin regenerative medicine.

### 3.3 *In vitro* anti-inflammatory activity of Do-EVLNs on LPS-Induced RAW 264.7 macrophages

**FIGURE 4:**
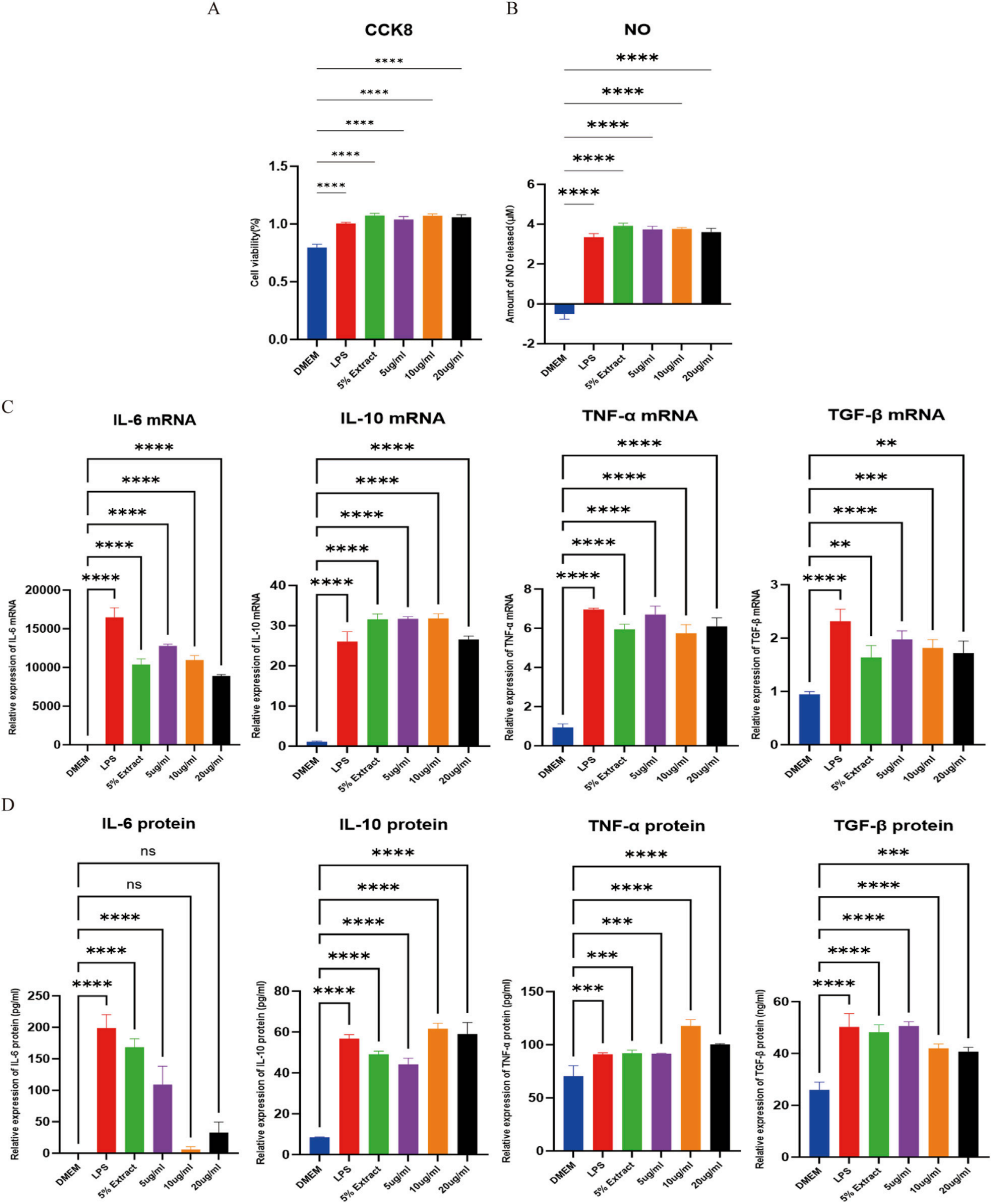
Study of *in vitro* anti-inflammatory activity. **(A)** Effects of Do-EVLNs on the viability of RAW 264.7 macrophages. **(B)** Effects of Do-EVLNs on nitric oxide (NO) secretion in LPS-stimulated RAW 264.7 macrophages. **(C)** Effects of Do-EVLNs on the mRNA expression levels of inflammatory genes in RAW 264.7 cells. **(D)** Effects of Do-EVLNs on inflammatory cytokine secretion in RAW 264.7 cells (*p* ≥ 0.05, ns; *p < 0.05, **p < 0.01, ***p < 0.001, and ****p < 0.0001).

To comprehensively evaluate the *in vitro* anti-inflammatory activity of Do-EVLNs, this study systematically investigated their effects on biosafety and the expression levels of associated inflammatory mediators in LPS-activated RAW 264.7 macrophages. Initial assessments using CCK-8 viability assays showed that Do-EVLNs exhibited no detectable cytotoxic effects at concentrations of 5–20 μg/mL after 24 h of incubation; all treatment groups maintained robust cellular viability, thus confirming the favorable biocompatibility of Do-EVLNs (Figure 4A). Concurrently, the quantification of extracellular NO release indicated no significant downregulation in NO secretion levels following Do-EVLN administration (Figure 4B).

Subsequent detailed profiling of the expression kinetics of representative inflammatory cytokines revealed that Do-EVLNs exhibit compelling anti-inflammatory activities at both transcriptional and translational levels. Stimulation with 1 μg/mL LPS markedly upregulated the mRNA expression of the pro-inflammatory genes IL-6 and TNF-α, as well as the anti-inflammatory genes IL-10 and TGF-β, validating the successful establishment of an *in vitro* macrophage inflammation model. Following intervention with Do-EVLNs, the transcription of the core pro-inflammatory gene IL-6 was significantly reversed, demonstrating a dose-dependent decline in its mRNA abundance. Concurrently, Do-EVLNs synergistically promoted the transcriptional upregulation of IL-10 mRNA. Furthermore, while reducing TNF-α gene expression, Do-EVLNs effectively countered and attenuated the elevated expression of TGF-β mRNA observed during inflammation (Figure 4C).

At the translational and secretory level, the aforementioned transcriptional cascades were further corroborated and amplified. In terms of pro-inflammatory cytokines, treatment with 10 or 20 μg/mL Do-EVLNs significantly suppressed IL-6 protein secretion, with expression levels approaching those of the DMEM vehicle control group. Notably, this inhibitory effect on IL-6 protein expression was significantly greater than that observed in the 5% Extract benchmark group. Simultaneously, Do-EVLNs facilitated a robust release of IL-10 protein, with the most pronounced upregulation occurring at a concentration of 10 μg/mL. The regulatory patterns observed for TNF-α and TGF-β protein expressions closely mirrored those seen at the transcriptional level (Figure 4D).

In conclusion, the consistent cascade of results across both nucleic acid and protein dimensions validates that Do-EVLNs exert anti-inflammatory efficacy by specifically suppressing the transcription and translation of IL-6 while simultaneously enhancing the transcription and expression of IL-10. These findings establish that Do-EVLNs possess significant *in vitro* anti-inflammatory capabilities.

### 3.4 Selective sorting of miRNAs into Do-EVLNs predicts the selective regulation of host inflammatory pathways

**FIGURE 5:**
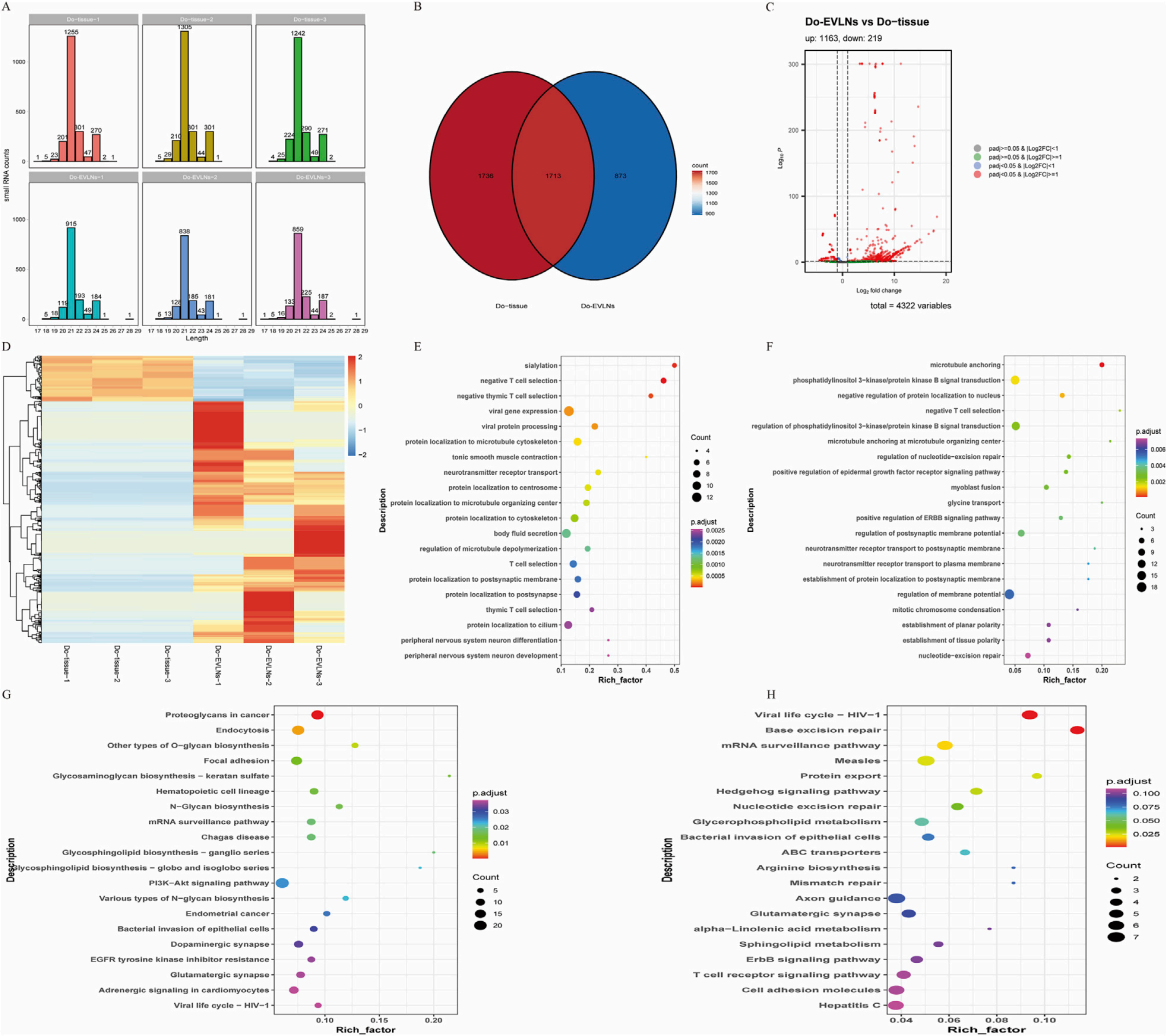
Characterization and differential expression of miRNAs in Do-EVLNs. **(A)** Length distribution of miRNAs in different samples. **(B)** Venn diagram showing distribution of miRNAs among different samples. **(C)** Volcano plot showing differential expression patterns between Do-EVLNs and Do-tissue. **(D)** Heatmap showing differentially expressed novel miRNAs between Do-EVLNs and Do-tissue. **(E)** GO classification of target genes of Do-EVLNs. **(F)** GO classification of target genes of Do-tissue. **(G)** KEGG enrichment analysis of target genes of Do-EVLNs. **(H)** KEGG enrichment analysis of target genes of Do-tissue (*p* ≥ 0.05, ns; *p < 0.05, **p < 0.01, ***p < 0.001, and ****p < 0.0001).

Small RNA profiling revealed a predominance of 21-nt isoforms in both groups (Figure 5A), with sequences exhibiting high evolutionary conservation across multiple plant species (Supplementary Figure S2). Differential expression analysis identified 1,163 upregulated and 219 downregulated miRNAs in Do-EVLNs compared with parental Do-tissue (Figures 5B–D). Notably, although miR-159 was more abundantly expressed in Do-tissue, specific miRNA families, including miR-399g-3p and miR-6300, were significantly enriched in Do-EVLNs, indicating a selective cargo-sorting mechanism. To explore their preliminary roles in biological regulation, potential human mRNA targets for these Do-EVLNs miRNAs were predicted. GO and KEGG pathway enrichment analyses indicated that these miRNAs are primarily linked to host immune and inflammatory pathways, including T-cell selection, cytoskeletal remodeling, and the PI3K/AKT signaling pathway (Figures 5E–H).

### 3.5 Transcriptomic profiling reveals distinct RNA cargo in Do-EVLNs compared to parental tissue

**FIGURE 6:**
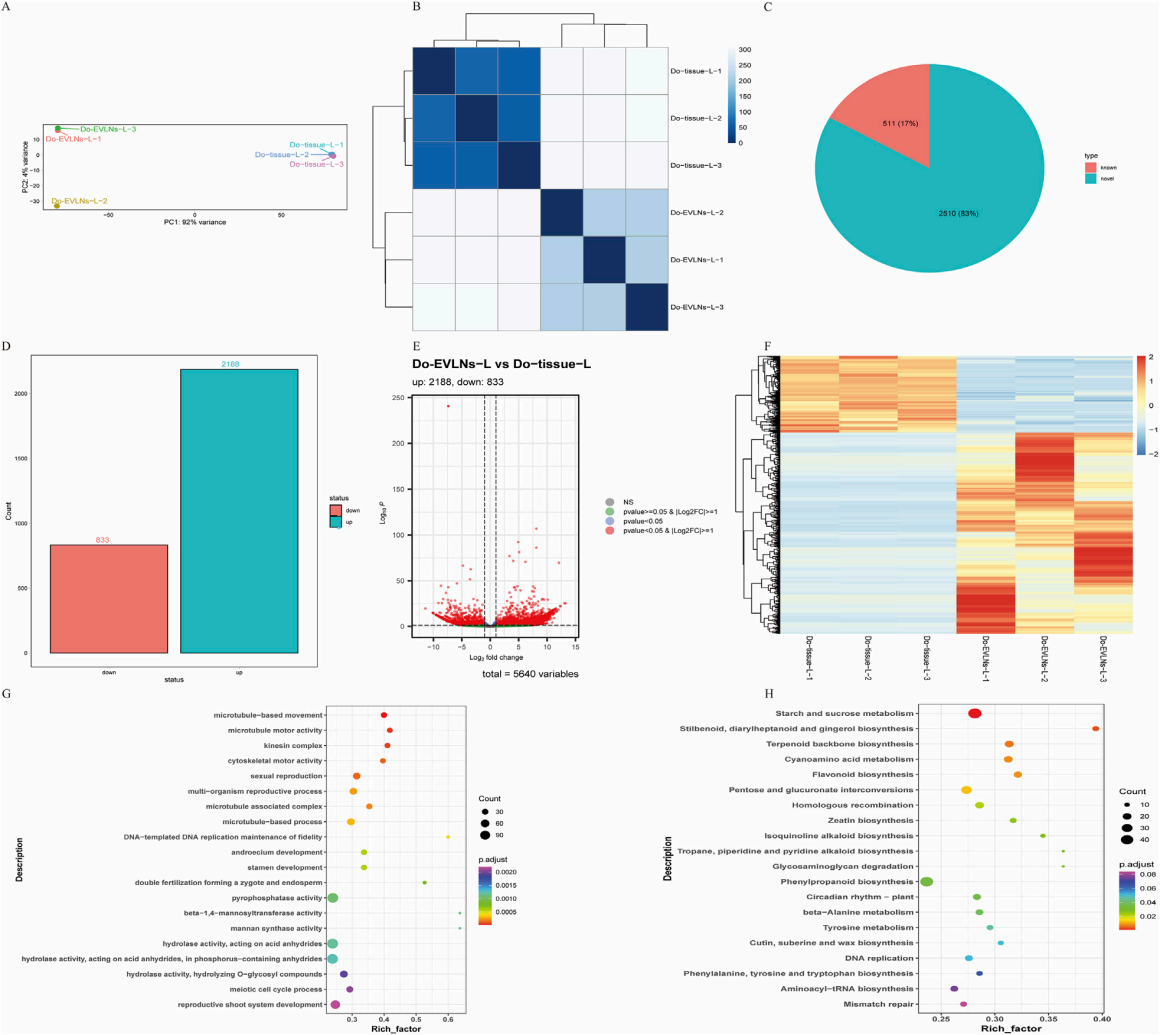
Characterization and differential expression of lncRNAs in Do-EVLNs. **(A)** PCA distribution of samples based on DESeq2 analysis. **(B)** Distance analysis of samples based on DESeq2. **(C)** Differential expression of known and novel lncRNAs across all samples. **(D)** Differential lncRNA expression between Do-EVLNs and Do-tissue. **(E)** Volcano plot showing differential expression patterns between Do-EVLNs and Do-tissue. **(F)** Heatmap showing differentially expressed novel lncRNAs between Do-EVLNs and Do-tissue. **(G)** GO classification of target genes of Do-EVLNs. **(H)** KEGG enrichment analysis of target genes of Do-EVLNs (*p* ≥ 0.05, ns; *p < 0.05, **p < 0.01, ***p < 0.001, and ****p < 0.0001).

**FIGURE 7:**
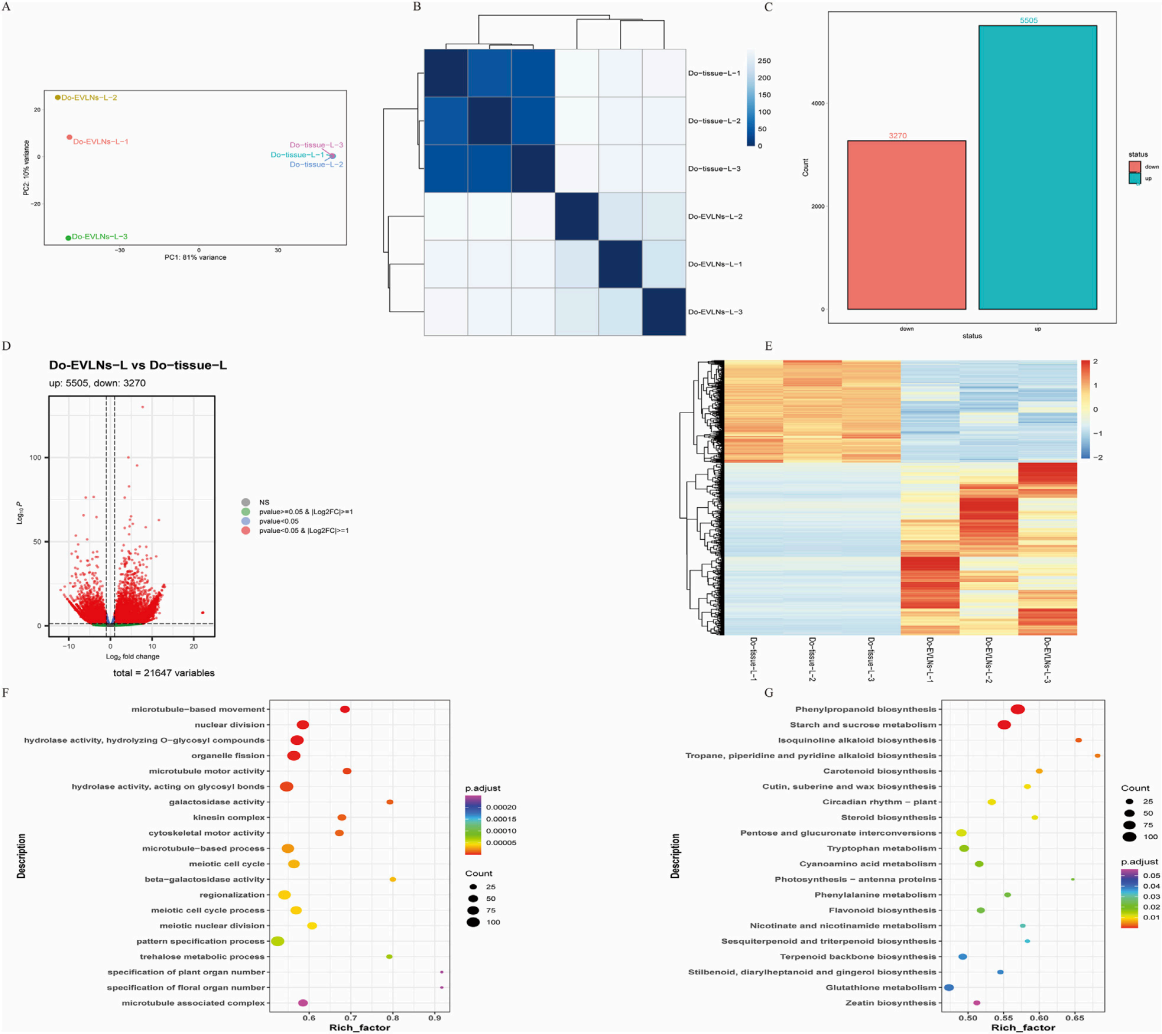
Characterization and differential expression of mRNAs in Do-EVLNs. **(A)** PCA distribution of samples based on DESeq2 analysis. **(B)** Distance analysis of samples based on DESeq2. **(C)** Differential mRNA expression between Do-EVLNs and Do-tissue. **(D)** Volcano plot showing differential expression patterns between Do-EVLNs and Do-tissue. **(E)** Heatmap showing differentially expressed novel mRNAs between Do-EVLNs and Do-tissue. **(F)** GO classification of target genes of Do-EVLNs. **(G)** KEGG enrichment analysis of target genes of Do-EVLNs (*p* ≥ 0.05, ns; *p < 0.05, **p < 0.01, ***p < 0.001, and ****p < 0.0001).

To comprehensively profile the nucleic acid cargo of Do-EVLNs and their parental tissue, strand-specific RNA sequencing was performed across small RNA, lncRNA, mRNA, and circRNA libraries. High-throughput sequencing generated a substantial volume of high-quality clean reads for downstream analysis (Supplementary Tables S1–S4). Principal component analysis (PCA) across all four transcriptomic datasets demonstrated a consistent pattern: biological replicates within each group clustered tightly, while the Do-tissue and Do-EVLNs groups displayed clear spatial segregation (Figures 6A,B; Figures 7A,B; Supplementary Figure S1). These results confirm robust intra-group reproducibility and highlight the selectively packaged RNA composition of Do-EVLNs, distinct from that of their parental tissue.

### 3.6 Characterization of long non-coding RNA cargo and functional annotation

In addition to miRNAs, Do-EVLNs encapsulated a complex array of long non-coding RNAs, with 3,021 identified long non-coding RNAs (lncRNAs), including 511 uncharacterized novel transcripts (Figure 6C). Comparative analysis showed 2,188 upregulated and 833 downregulated lncRNAs in Do-EVLNs relative to the parental Do-tissue group (Figures 6D–F). To elucidate the synergistic regulatory network of Do-EVLN cargo, functional enrichment analyses were conducted on the predicted target genes of these lncRNAs. GO and KEGG pathway enrichment analyses highlighted key biological processes associated with tissue homeostasis and inflammation, notably cytoskeletal microtubule dynamics, intracellular hydrolase activity, and metabolic reprogramming (including starch, sucrose, tryptophan metabolism, and flavonoid biosynthesis) (Figures 6G,H).

### 3.7 mRNA profiling and integrated molecular enrichment

Transcriptomic analysis of mRNA cargo identified 8,775 differentially expressed mRNAs (5,505 upregulated and 3,270 downregulated) in Do-EVLNs relative to the parental Do-tissue group (Figures 7C–E). Functional enrichment analyses of these differentially expressed mRNA profiles further elucidated the underlying molecular mechanisms. Consistent with the non-coding RNA landscape, structural and metabolic pathways were highly enriched, demonstrating a coordinated response targeting host cellular metabolism and structural remodeling (Figures 7F,G). This integrated transcriptomic landscape suggests that Do-EVLNs may exert anti-inflammatory and tissue-repair effects by co-delivering a multifaceted RNA cargo.

### 3.8 Specialized landscape of circular RNA cargo in Do-EVLNs

**FIGURE 8:**
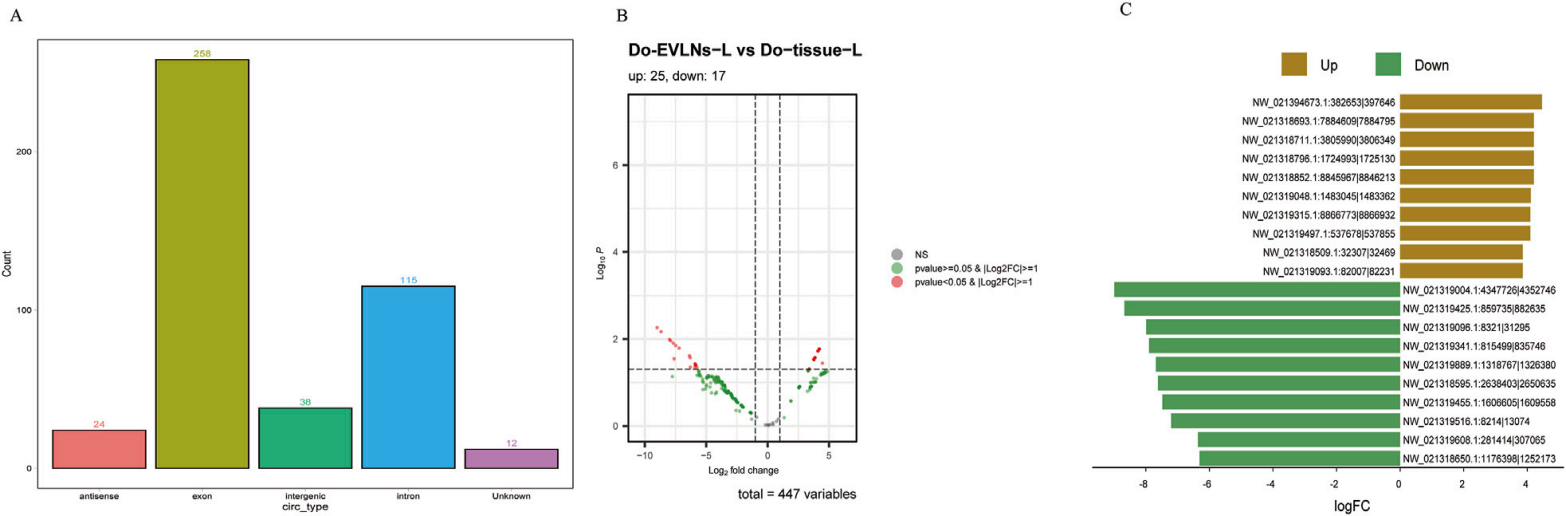
Characterization and differential expression of circRNAs in Do-EVLNs. **(A)** CircRNA expression in all samples. **(B)** Volcano plot showing differential expression patterns between Do-EVLNs and Do-tissue. **(C)** Inverted bar chart showing differential expression of circRNAs between Do-EVLNs and Do-tissue (*p* ≥ 0.05, ns; *p < 0.05, **p < 0.01, ***p < 0.001, and ****p < 0.0001).

Circular RNA (CircRNA) profiling was conducted to thoroughly assess the non-coding RNA spectrum within Do-EVLNs. A total of 447 circRNAs were identified, predominantly of exonic origin (Figure 8A). Comparative profiling revealed a distinctive expression signature, with 25 circRNAs significantly enriched and 17 significantly depleted in Do-EVLNs relative to the parental Do-tissue group (Figure 8B). To gain deeper insights into this specialized cargo, the top differentially expressed circular transcripts were characterized based on their genomic loci and host gene annotations (Figure 8C). Notably, several highly enriched circRNAs (e.g., NW_021394673.1:382,653|397,646 and NW_021318693.1:7884609|7884795) were transcribed from host genes functionally linked to plant stress adaptation, carbohydrate metabolism, and cell wall remodeling. This selective enrichment suggests that specific circRNA species are preferentially sorted into Do-EVLNs, potentially serving as stable regulatory elements during biological processes.

### 3.9 miRNA expression validation by RT-qPCR

**FIGURE 9:**
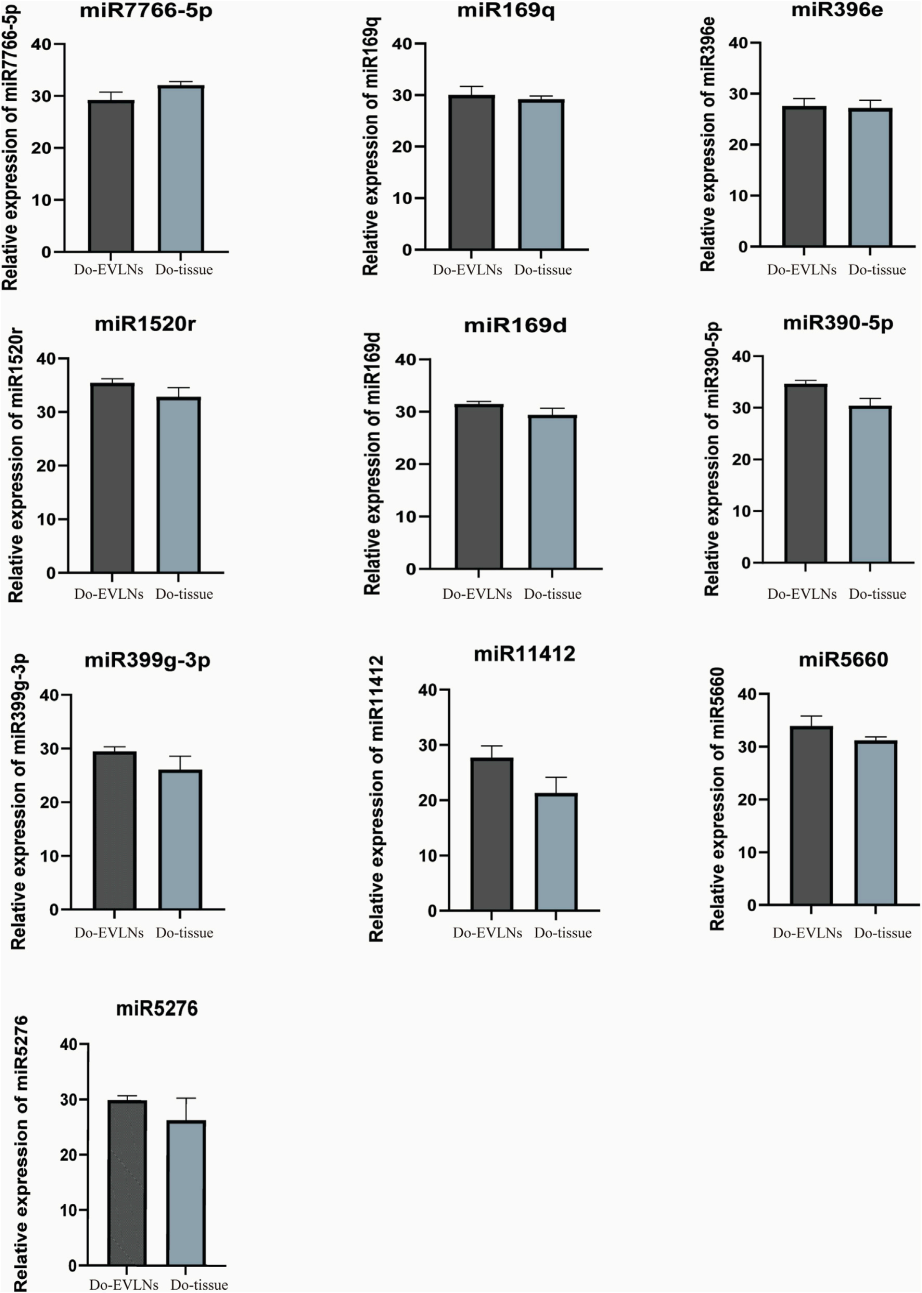
Q-PCR validation of differentially expressed miRNAs between Do-EVLNs and Do-tissue (*p* ≥ 0.05, ns, *p < 0.05, **p < 0.01, ***p < 0.001, and ****p < 0.0001).

To validate the small RNA sequencing (sRNA-seq) profiles, ten candidate miRNAs underwent RT-qPCR analysis (Figure 9). The validation results corroborated the transcriptomic data, confirming the upregulation of nine miRNAs and downregulation of one miRNA in Do-EVLNs compared to the parental Do-tissue group.

Functional annotation based on existing literature indicates that these enriched miRNAs hold significant potential for biological regulation in mammals. Specifically, these candidates are implicated in modulating a diverse range of critical physiological and pathological processes in mammals, including cardiovascular and immune homeostasis (e.g., the miR-169 family) (Li et al., 2025), lipid metabolism (miR-396e) (Bian et al., 2023), angiogenesis (miR-223–5p) (Li et al., 2023), renal function (miR-399g-3p) (Liang et al., 2025), cellular stress responses (e.g., miR-11412) (Byrd and Brewer, 2013), and neurodevelopment (miR-7766–5 and miR-390–5p) (Sing et al., 2021; Lopizzo et al., 2019). While these literature-based insights highlight the broad therapeutic potential of Do-EVLNs, further experimental elucidation is required to identify the precise molecular targets and signaling axes in human cells in future studies.

## 4 Discussion

This study presents a comprehensive characterization and functional elucidation of Do-EVLNs. Experimental results demonstrate that Do-EVLNs exhibit classical vesicular morphology with a mean hydrodynamic diameter of 132.1 ± 1.9 nm and a negative zeta potential of −24 ± 0.16 mV. This negative surface charge is statistically verified to provide optimal colloidal stability in suspension, while their nanoscale dimensions align precisely with the classic size-range typically associated with energy-dependent endocytic pathways. Importantly, *in vitro* biological evaluations confirm that Do-EVLNs possess a unique dual-functional repertoire, modulating both tissue-regenerative phenotypes in skin-resident cells and anti-inflammatory responses in macrophages—all without detectable cytotoxicity at the tested dosages.

Functionally, the administration of Do-EVLNs promotes proliferation, migration, and angiogenesis while attenuating apoptosis in human skin-resident and endothelial cells (HUVEC, HACAT, and BJ-1). These regenerative effects are substantiated by cell-cycle analysis, which reveals an accelerated G1-to-S phase transition. To explore their immunomodulatory capacity, Do-EVLNs were evaluated in an *in vitro* inflammation model using LPS-activated RAW 264.7 macrophages. At concentrations of 5–20 μg/mL, Do-EVLNs exhibited favorable biosafety while effectively intercepting the localized inflammatory cascade. This was evidenced by a prominent, dose-dependent reversal of the core pro-inflammatory mediator IL-6 at both transcriptional and translational levels, with protein secretion levels returning close to baseline vehicle control. Notably, the inhibitory efficacy against IL-6 protein expression surpassed that of the raw 5% Extract benchmark, confirming that the vesicle-encapsulated form enhances biological potency compared to non-fractionated soluble plant constituents. Additionally, the modulation of the cytokine network—where Do-EVLNs drove the upregulation of the anti-inflammatory cytokine IL-10 while counteracting the elevation of TNF-α and TGF-β transcripts—demonstrates the capacity of Do-EVLNs to suppress specific macrophage inflammatory pathways while promoting skin-resident cell migration.

To investigate the underlying molecular mechanisms, comprehensive transcriptomic profiling revealed a highly regulated packaging of RNA cargoes (miRNAs, lncRNAs, mRNAs, and circRNAs) distinct from the parental tissue. Bioinformatic functional enrichment analyses of this sequencing dataset indicate potential involvement of the PI3K/AKT signaling pathway, endocytic trafficking, and cytoskeletal remodeling (Smillie et al., 2019; Yu et al., 2024). However, these identified pathways remain theoretical predictions that require independent biochemical validation. Moreover, while the medicinal lineage of *D. officinale* suggests that these nanovesicles may encapsulate bioactive macromolecules such as specific polysaccharides, alkaloids, or flavonoids, their direct role as co-deliverers of biological signals alongside the investigated RNAs remains a hypothesis, as intra-vesicular proteomic and metabolomic profiles were not quantified in this study. Whether these unquantified components can directly scavenge reactive oxygen species (ROS) or whether endogenous plant proteins (such as lectins or heat shock proteins) can bind to mammalian surface receptors remains speculative and awaits confirmation through further specific experiments.

Bioinformatically, the enrichment of immune-related transcripts and flavonoid biosynthesis pathways correlates evolutionarily with specialized secondary metabolic networks (Frühbeis et al., 2013; Liu et al., 2022). This sequencing repertoire distinguishes Do-EVLNs from the transcriptomic profiles reported for common edible plant vesicles (such as ginger, grape, or citrus), which primarily modulate intestinal homeostasis. While it is tempting to hypothesize that the selective packaging of RNA cargo reflects its distinct medicinal lineage under specific environmental stressors, further comparative studies are necessary to establish a causal link between the evolutionary lineage of the orchid and its multi-target tissue repair capacities.

Notably, the relative expression trends of ten representative transcripts, including the miR-169 family and miR-223–5p, were evaluated via RT-qPCR. Although these target validation assays confirmed consistent expression directionality aligning with our sequencing dataset, relative changes did not achieve statistical significance (*p* < 0.05). Consequently, while existing literature recognizes the miR-169 family and miR-223–5p as master regulators of macrophage polarization and vascular homeostasis, their roles as primary functional effectors driving the observed IL-6 suppression and IL-10 elevation in our assays remain preliminary correlations rather than established causal mechanisms.

Our *in vitro* and transcriptomic data provide a rigorous foundational characterization of Do-EVLNs. Future *in vivo* studies utilizing animal models of inflammatory skin disorders are indispensable to definitively test these predicted functional pathways and fully establish their therapeutic potential.

## 5 Conclusion

This study characterized Do-EVLNs with a verified mean diameter of 132.1 ± 1.9 nm and negative zeta potential of −24 ± 0.16 mV. The findings demonstrated their dual *in vitro* biological activities across both human skin-resident cells and murine macrophages. The ability of Do-EVLNs to accelerate cellular proliferation and migration—evidenced by an observed G1-to-S phase cell-cycle transition—alongside their modulation of localized inflammatory responses via IL-6 suppression and IL-10 upregulation provides clear experimental evidence of multi-target effects. Although these consistent cellular and cytokine cascades present definitive phenotypic profiles under *in vitro* conditions, the precise molecular components and potential regulatory mechanisms driving these events remain unverified predictions within the current dataset. Future *in vivo* studies utilizing animal models are crucial to determine whether these observed effects are translatable to complex physiological environments and to elucidate the specific causal pathways governing tissue repair. Ultimately, these empirical data establish the physicochemical and *in vitro* functional profiles of Do-EVLNs, marking them as promising bioactive nanoplatforms for further anti-inflammatory and dermatological investigations.

## Data Availability

The datasets presented in this study can be found in online repositories. The names of the repository/repositories and accession number(s) can be found in the article/Supplementary Material.

## References

[B1] Bellucci M. Pompa A. De Marcos Lousa C. Panfili E. Orecchini E. Maricchiolo E. (2021). Human indoleamine 2,3-dioxygenase 1 (IDO1) expressed in plant cells induces kynurenine production. Int. J. Mol. Sci. 22, 5102. 10.3390/ijms22105102 34065885 PMC8151846

[B2] Bian Y. Li W. Jiang X. Yin F. Yin L. Zhang Y. (2023). Garlic-derived exosomes carrying miR-396e shapes macrophage metabolic reprograming to mitigate the inflammatory response in Obese adipose tissue. J. Nutr. Biochem. 113, 109249. 10.1016/j.jnutbio.2022.109249 36496060

[B3] Bruno S. P. Paolini A. D'Oria V. Sarra A. Sennato S. Bordi F. (2021). Extracellular vesicles derived from citrus sinensis modulate inflammatory genes and tight junctions in a human model of intestinal epithelium. Front. Nutr. 8, 778998. 10.3389/fnut.2021.778998 34901124 PMC8652296

[B4] Byrd A. E. Brewer J. W. (2013). Micro(RNA)managing endoplasmic reticulum stress. IUBMB Life 65, 373–381. 10.1002/iub.1151 23554021 PMC3637854

[B5] Cai Z. Lu C. Chen D. Zhang S. Zhou J. Yu S. (2025). Wogonin modulates macrophage polarization and inflammatory signaling through the LSD1-p65 axis to alleviate osteoarthritis. Phytomedicine 146, 157149. 10.1016/j.phymed.2025.157149 40818185

[B6] De Marchis F. Vanzolini T. Maricchiolo E. Bellucci M. Menotta M. Di Mambro T. (2024). A biotechnological approach for the production of new protein bioplastics. Biotechnol. J. 19, e2300363. 10.1002/biot.202300363 37801630

[B7] Frühbeis C. Fröhlich D. Kuo W. P. Amphornrat J. Thilemann S. Saab A. S. (2013). Neurotransmitter-triggered transfer of exosomes mediates oligodendrocyte-neuron communication. PLoS Biol. 11, e1001604. 10.1371/journal.pbio.1001604 23874151 PMC3706306

[B8] Kim J. Li S. Zhang S. Wang J. (2022). Plant-derived exosome-like nanoparticles and their therapeutic activities. Asian J. Pharm. Sci. 17, 53–69. 10.1016/j.ajps.2021.05.006 35261644 PMC8888139

[B9] Li S. Yang K. Cao W. Guo R. Liu Z. Zhang J. (2023). TanShinone IIA enhances the therapeutic efficacy of mesenchymal stem cells derived exosomes in myocardial ischemia/reperfusion injury via up-regulating miR-223-5p. J. Control. Release 358, 13–26. 10.1016/j.jconrel.2023.04.014 37086952

[B10] Li S. Liu F. Zhang S. Sun X. Li X. Yue Q. (2025). Lavender exosome-like nanoparticles attenuate UVB-Induced photoaging via miR166-mediated inflammation and collagen regulation. Sci. Rep. 15, 21286. 10.1038/s41598-025-08817-2 40596624 PMC12216414

[B11] Liang L. Lv J. Li W. Song C. Chen Y. Wei H. (2025). Reduced miR-338-3p contributes to polycystic ovarian syndrome by inhibiting proliferation and enhancing apoptosis. Hereditas 162, 126. 10.1186/s41065-025-00498-1 40652291 PMC12254983

[B12] Liu S. Zhang H. Yuan Y. (2022). A comparison of the flavonoid biosynthesis mechanisms of dendrobium species by analyzing the transcriptome and metabolome. Int. J. Mol. Sci. 23, 11980. 10.3390/ijms231911980 36233278 PMC9569625

[B13] Liu X. Lou K. Zhang Y. Li C. Wei S. Feng S. (2024). Unlocking the medicinal potential of plant-derived extracellular vesicles: current progress and future perspectives. Int. J. Nanomedicine 19, 4877–4892. 10.2147/IJN.S463145 38828203 PMC11141722

[B14] Lopizzo N. Zonca V. Cattane N. Pariante C. M. Cattaneo A. (2019). miRNAs in depression vulnerability and resilience: novel targets for preventive strategies. J. Neural Transm. (Vienna) 126, 1241–1258. 10.1007/s00702-019-02048-2 31350592 PMC6746676

[B15] Maricchiolo E. Creanza P. Osnato M. Tiboni M. Casettari C. Aluigi A. (2026). Plant vs mammal extracellular vesicles: new tools in therapeutic drug delivery. Curr. Res. Biotechnol. 11, 100352. 10.1016/j.crbiot.2025.100352

[B16] Nemati M. Singh B. Mir R. A. Nemati M. Babaei A. Ahmadi M. (2022). Plant-derived extracellular vesicles: a novel nanomedicine approach with advantages and challenges. Cell Commun. Signal. 20, 69. 10.1186/s12964-022-00889-1 35606749 PMC9128143

[B17] Singh G. Storey K. B. (2021). MicroRNA cues from nature: a roadmap to decipher and combat challenges in human health and disease? Cells 10, 3374. 10.3390/cells10123374 34943882 PMC8699674

[B18] Smillie C. S. Biton M. Ordovas-Montanes J. Sullivan K. M. Burgin G. Graham D. B. (2019). Intra- and inter-cellular rewiring of the human colon during ulcerative colitis. Cell 178, 714–730. 10.1016/j.cell.2019.06.029 31348891 PMC6662628

[B19] Tang H. Zhao T. Sheng Y. Zheng T. Fu L. Zhang Y. (2017). Dendrobium officinale Kimura et Migo: a review on its ethnopharmacology, phytochemistry, pharmacology, and industrialization. Evid. Based Complement. Altern. Med. 2017, 7436259. 10.1155/2017/7436259 28386292 PMC5366227

[B20] Teng Y. He J. Zhong Q. Zhang Y. Lu Z. Guan T. (2022). Grape exosome-like nanoparticles: a potential therapeutic strategy for vascular calcification. Front. Pharmacol. 13, 1025768. 10.3389/fphar.2022.1025768 36339605 PMC9634175

[B21] Teng Y. Luo C. Qiu X. Mu J. Sriwastva M. K. Xu Q. (2025). Plant-nanoparticles enhance anti-PD-L1 efficacy by shaping human commensal microbiota metabolites. Nat. Commun. 16, 1295. 10.1038/s41467-025-56498-2 39900923 PMC11790884

[B22] Xu X. Zhang C. Wang N. Xu Y. Tang G. Xu L. (2022). Bioactivities and mechanism of actions of dendrobium officinale: a comprehensive review. Oxid. Med. Cell Longev. 2022, 6293355. 10.1155/2022/6293355 36160715 PMC9507758

[B23] Yan L. Cao Y. Hou L. Luo T. Li M. Gao S. (2025). Ginger exosome-like nanoparticle-derived miRNA therapeutics: a strategic inhibitor of intestinal inflammation. J. Adv. Res. 69, 1–15. 10.1016/j.jare.2024.04.001 38588850 PMC11954804

[B24] Yu Z. Yong Y. Liu X. Ma X. Abd El-Aty A. M. Li L. (2024). Insights and implications for transcriptomic analysis of heat stress-induced intestinal inflammation in pigs. BMC Genomics 25, 1110. 10.1186/s12864-024-10928-5 39563245 PMC11577645

[B25] Zhao B. Lin H. Jiang X. Li W. Gao Y. Li M. (2024). Exosome-like nanoparticles derived from fruits, vegetables, and herbs: innovative strategies of therapeutic and drug delivery. Theranostics 14, 4598–4621. 10.7150/thno.97096 39239509 PMC11373634

