# Supplementary material for: Dendrobium officinale nanovesicles: transcriptomic landscape and anti-inflammatory roles
Source: Front Bioeng Biotechnol. 2026 Jul 29;14:1848009. doi: 10.3389/fbioe.2026.1848009 (PMC13462397; doi:10.3389/fbioe.2026.1848009)
Supplement: Supplementary file 1 [file Supplementaryfile1.pdf]

Supplementary Tables 1: RNA sequencing result

Class	reads_raw	Q20_raw	Q30_raw	raw_GC%	reads_clean	Q20_clean	Q30_clean	clean_GC%
Do-tissue-1_R1	12523934	87.70%	71.71%	62%	12429967	99.35%	97.36%	49%
Do-tissue-1_R2	12523934	90.19%	77.13%	59%	12429967	99.50%	98.16%	49%
Do-tissue-2_R1	10304730	88.51%	72.99%	63%	10225262	99.41%	97.53%	49%
Do-tissue-2_R2	10304730	90.40%	77.54%	58%	10225262	99.54%	98.30%	49%
Do-tissue-3_R1	11841907	86.64%	69.41%	59%	11754418	99.40%	97.49%	50%
Do-tissue-3_R2	11841907	88.67%	74.13%	57%	11754418	99.52%	98.22%	50%
Do-EVLNs-1_R1	17055173	88.02%	74.61%	57%	16911661	99.18%	97.07%	50%
Do-EVLNs-1_R2	17055173	90.16%	77.37%	54%	16911661	99.58%	98.40%	50%
Do-EVLNs-2_R1	15676928	86.23%	72.58%	54%	15549206	99.35%	97.43%	50%
Do-EVLNs-2_R2	15676928	89.62%	76.53%	55%	15549206	99.61%	98.52%	50%
Do-EVLNs-3_R1	16461257	87.13%	73.05%	56%	16323197	99.31%	97.31%	50%
Do-EVLNs-3_R2	16461257	88.28%	74.68%	54%	16323197	99.58%	98.40%	50%

Supplementary Figure 1: miRNA PCA and sample distance analysis

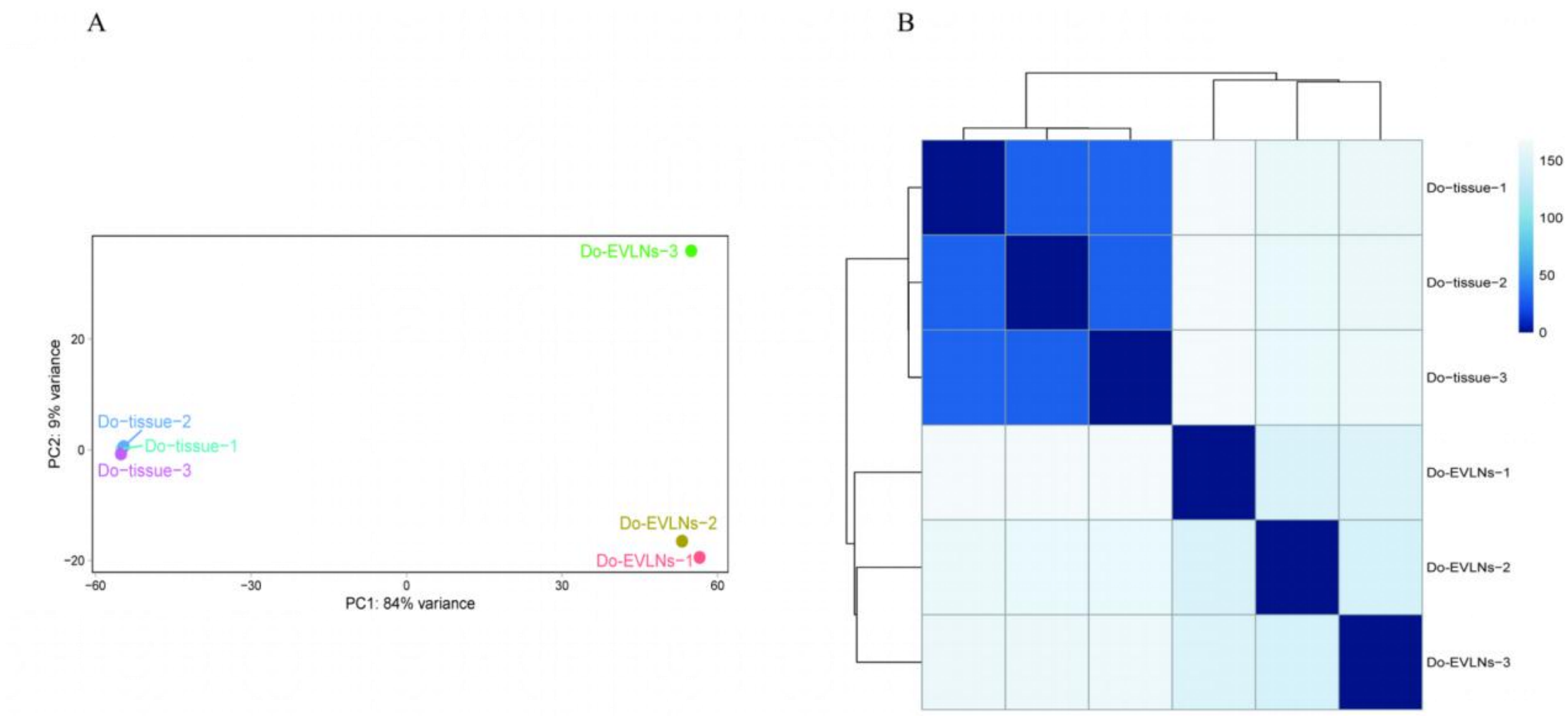

Clustering of different samples. (A) PCA distribution of samples based on DESeq2. (B) Distance analysis of samples based on DESeq2.

Supplementary Figure 2: Results of miRNA species similarity analysis

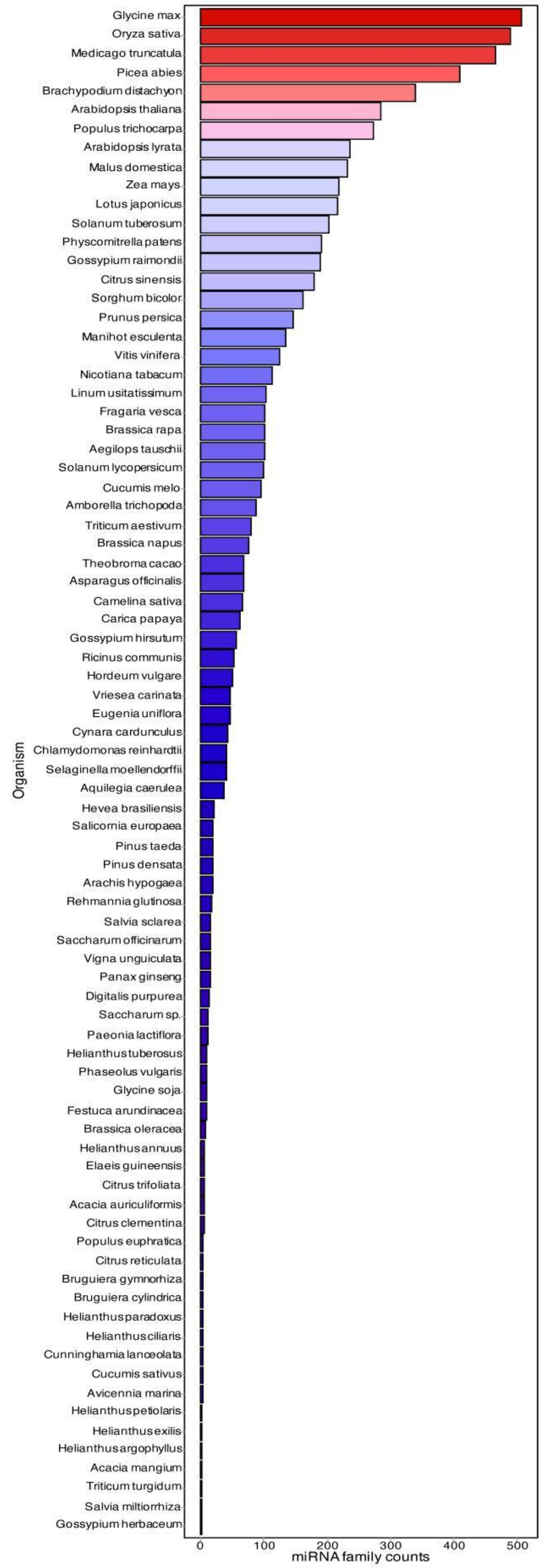

Supplementary Figure 3: Reproducibility experiments of Do-EVLNs uptake in *vitro*

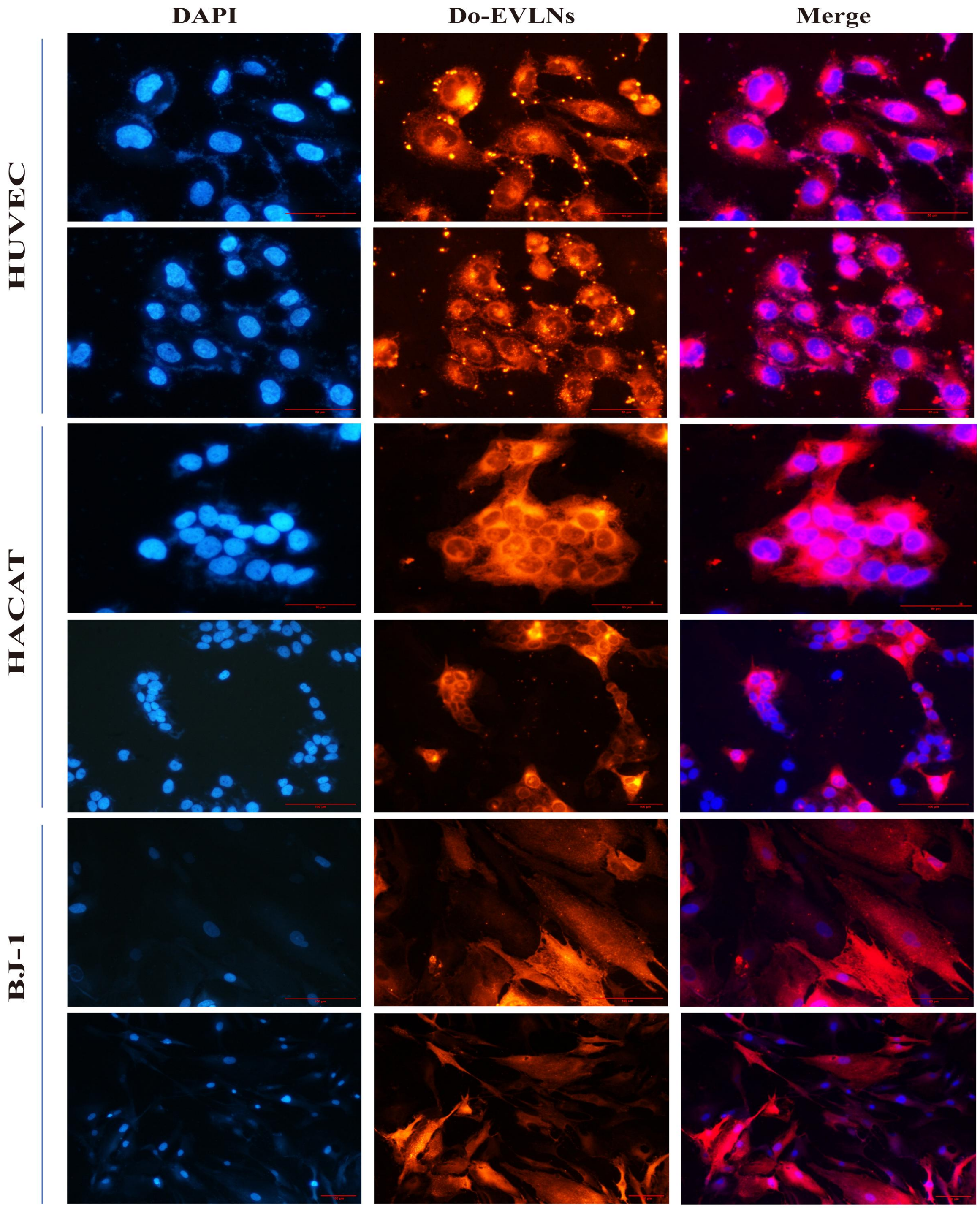

Representative immunofluorescence images validating the reproducibility of Do-EVLN uptake in HUVEC, HACAT, and BJ-1 cells. Scale bar specifications for each cell group: HUVEC (Scale bar = 50 μm); HACAT (top row = 50 μm; bottom row = 100 μm); BJ-1 (Scale bar = 100 μm).

Supplementary Tables 2: Length distribution of miRNAs

Length	Do-tissue-1	Do-tissue-2	Do-tissue-3	Do-EVLNs-1	Do-EVLNs-2	Do-EVLNs-3
17	617464	559968	624076	1453953	1251963	1385643
22	1452555	1418291	1518051	2706719	2411951	2574734
27	737081	647847	774115	2709997	2527478	2624633
32	660763	607784	754861	3447673	3284485	3407961
37	250329	228871	276704	1449694	1480856	1434741
42	169590	145168	180271	588186	623228	584914
47	92081	78939	100160	335035	363090	331756
52	53928	46292	58959	237828	257502	226960
57	57363	45924	58500	146458	155665	134736
62	27935	22853	30406	90380	101702	89047
67	17478	14269	18776	46826	52628	43562
72	17955	14587	17988	30601	35505	28097
77	34187	27717	34704	22277	25228	19977
82	20573	17261	21941	17310	16379	13799
87	18611	14690	19628	12176	13311	10548
92	9701	7530	9841	9974	10882	8484
97	9853	7697	10305	9178	10072	7796
102	4305	3281	4389	7757	8543	6495
107	3592	2619	3586	5905	6573	4773
112	3999	3041	4202	4961	5265	4097
117	15446	10000	14560	5056	5599	4176
122	48841	30337	46519	4982	5633	4199
127	6800	4843	7163	1782	1797	1452

132	6598	4574	6631	1680	1886	1422
137	2923	2042	3079	1233	1499	1177
142	3066	2277	3246	846	965	733
146	84	51	66	27	48	29
150	319916	230122	356792	5640	6140	5013

Supplementary Tables 3: Comparative analysis of miRNA abundance among different sample

representive_family	miRNA	total	Do-tissue-1	Do-tissue-2	Do-tissue-3	Do-EVLNs-1	Do-EVLNs-2	Do-EVLNs-3
miR159	mir1	70974	23124	23821	23618	144	122	145
miR159a	mir2	70965	23116	23820	23618	144	122	145
miR159f-3p	mir3	70944	23113	23806	23614	144	122	145
miR159b-3p.1	mir4	69938	22786	23455	23287	143	122	145
miR159a	mir5	69931	22780	23454	23287	143	122	145
miR159a	mir6	69929	22778	23454	23287	143	122	145
miR159j-3p	mir7	69908	22775	23440	23283	143	122	145
miR319k	mir8	26109	7947	9616	8515	7	7	17
miR319a	mir9	26109	7948	9616	8514	7	7	17
miR319j	mir10	26109	7948	9616	8514	7	7	17

Supplementary Tables 4: Sequencing statistics of lncRNAs

Class	reads_raw	Q20_raw	Q30_raw	raw_GC%	reads_clean	Q20_clean	Q30_clean	clean_GC%
Do-EVLNs-L-1_R1	36721610	89.91%	80.27%	50%	32644913	96.12%	91.41%	48%
Do-EVLNs-L-1_R2	36721610	92.18%	86.35%	50%	32644913	96.67%	93.22%	48%
Do-EVLNs-L-2_R1	35536074	91.96%	84.09%	50%	32200275	96.69%	92.42%	49%
Do-EVLNs-L-2_R2	35536074	93.75%	88.97%	50%	32200275	97.37%	94.51%	49%
Do-EVLNs-L-3_R1	36709396	92.01%	84.17%	49%	33353240	96.74%	92.49%	49%
Do-EVLNs-L-3_R2	36709396	93.86%	89.09%	50%	33353240	97.38%	94.48%	49%
Do-tissue-L-1_R1	32976252	96.24%	91.53%	52%	32268224	98.46%	95.46%	52%
Do-tissue-L-1_R2	32976252	98.17%	95.95%	52%	32268224	99.01%	97.29%	52%
Do-tissue-L-2_R1	33913388	96.08%	91.13%	50%	33064477	98.30%	95.14%	50%
Do-tissue-L-2_R2	33913388	98.00%	95.78%	51%	33064477	98.96%	97.27%	50%
Do-tissue-L-3_R1	39110204	96.18%	91.55%	51%	38183056	98.46%	95.55%	51%
Do-tissue-L-	39110	98.02	95.68	51%	3818305	98.94	97.13	51%

3_R2	204	%	%	6	%	%
